# In Vitro Trypanocidal Activity of Antibodies to Bacterially Expressed *Trypanosoma brucei* Tubulin

**Published:** 2012

**Authors:** DP Kateete, C Alezuyo, A Nanteza, C Asiimwe, GW Lubega

**Affiliations:** 1 School of Biomedical Sciences, Makerere University College of Health Sciences, Kampala, Uganda; 2 Family planning and HIV Programs, P.O Box 26 Mubende, Uganda; 3 College of Veterinary Medicine, Animal Resources & Biosecurity, Makerere University, Kampala, Uganda; 4 Foundation for Innovative New Diagnostics (FIND), Kampala, Uganda

**Keywords:** Alpha tubulin, Beta tubulin, *Trypanosoma brucei*, Protein expression, Gene cloning

## Abstract

**Background:**

There are only four drugs for treating African trypanosomiasis, a devastating disease in sub-Saharan Africa. With slow discovery of better drugs, vaccination is viewed as the best method of control. We previously showed that antibodies to native *Trypanosoma brucei brucei* tubulin inhibit the growth of trypanosomes in culture. Here, we aimed to determine the effect of antibodies to bacterially expressed trypanosome tubulin on *T. brucei brucei* growth.

**Methods:**

*T. brucei brucei* alpha and beta tubulin genes were individually expressed in *Escherichia coli* under the tryptophan promoter. Monoclonal tubulin antibodies reacted specifically with the expressed tubulins with no cross-reaction with the opposite tubulin. Rabbits were immunized with 450µg each of the concentrated recombinant tubulin, and production of antibodies assessed by ELISA and Western blotting. The effect of polyclonal antibodies on trypanosome growth was determined by culturing bloodstream *T. brucei brucei* in up to 25% of antisera.

**Results:**

Low antisera dilutions (25%) from the immunized rabbits inhibited trypanosome growth. The most cytotoxic antisera were from one rabbit immunized with a mixture of both alpha and beta tubulins. However, the result was not reproduced in other rabbits and there was no apparent effect on growth at higher antisera dilutions.

**Conclusion:**

Antibodies to bacterially expressed trypanosome tubulin are not effective at killing cultured bloodstream trypanosomes.

## Introduction

African trypanosomiasis (Sleeping sickness in humans and Nagana in livestock), a devastating disease in sub-Saharan Africa, is caused by African trypanosomes. The latter are protozoan haemoflagellates transmitted by tsetse flies of the genus Glossina. Sleeping sickness is geographically stratified with a mild form in West Africa caused by *Trypanosoma brucei gambiense* while a severe form caused by *T. b. rhodesiense* occurs in East and Southern Africa. However, both subtypes of disease occur concurrently in Uganda; *T. b. gambiense* disease affects the North-Western part of the country (West Nile) (1) while *T. b. rhodesiense* commonly occurs in South-Eastern Uganda (1, 2).

The tsetse flies infest about 10 million square km of arable land in Africa, hindering livestock production and worsening malnutrition. Chemotherapy the mainstay of control relies on only four drugs which tend to be toxic to patients (3, 4). Currently there's no vaccine against African trypanosomiasis. Antigenic variation, a phenomenon in which trypanosomes alter their variant surface glycoproteins (VSG) on a regular basis paints a gloomy picture for vaccine development; as soon as the host mounts an immune response against one VSG, the expression pattern changes and a strain of parasite emerges that escapes host immunity (5).

Since discovery of more efficacious drugs is slow, vaccination is viewed as the most promising and sustainable method of controlling African trypanosomiasis (6, 7, 8). As such, several groups have explored the possibility that an effective anti-trypanosome vaccine can be developed (9). Events in the vector or hosts suffering from the disease suggest a possibility of immunity and these may form a basis for vaccine development (8, 10, 11). Vaccine design strategies have focused on the invariant surface glycoproteins (IVGs), flagellar pocket proteins, cyteine proteinases (congopain) and intracellular antigens such as the microtubule associated proteins (MAPs) and tubulin (12, 13, 14).

We previously demonstrated that immunization with native *T. brucei brucei* microtubule extracts protects mice against trypanosomiasis (15), and that rabbit antibodies to tubulin-rich fractions from *T. brucei brucei* inhibit the growth of trypanosomes in culture (16). Earlier, other groups had demonstrated that flagella pocket antigens (which contain microtubule components) protect against trypanosome infections in laboratory and large animals (13), while MAPs completely protected rats and mice against trypanosomiasis (14, 17).

For detailed studies however, obtaining ample amounts of native tubulin-rich fractions from trypanosomes is challenging. As such, several groups have resorted to recombinant trypanosome tubulin isoforms expressed in *Escherichia coli* (20) . While it has been demonstrated that immunization with recombinant β-tubulin expressed in *E. coli* (18) partially protects against *T. evansi, T. equiperdum* and *T. b. brucei* infections in mice, it has not been determined whether those antibodies can as well kill trypanosomes in culture. This study therefore aimed to determine the effect of antibodies to bacterially expressed tubulin on trypanosome growth.

## Materials and Methods

### T. brucei tubulin clones

**Fig. 1:**
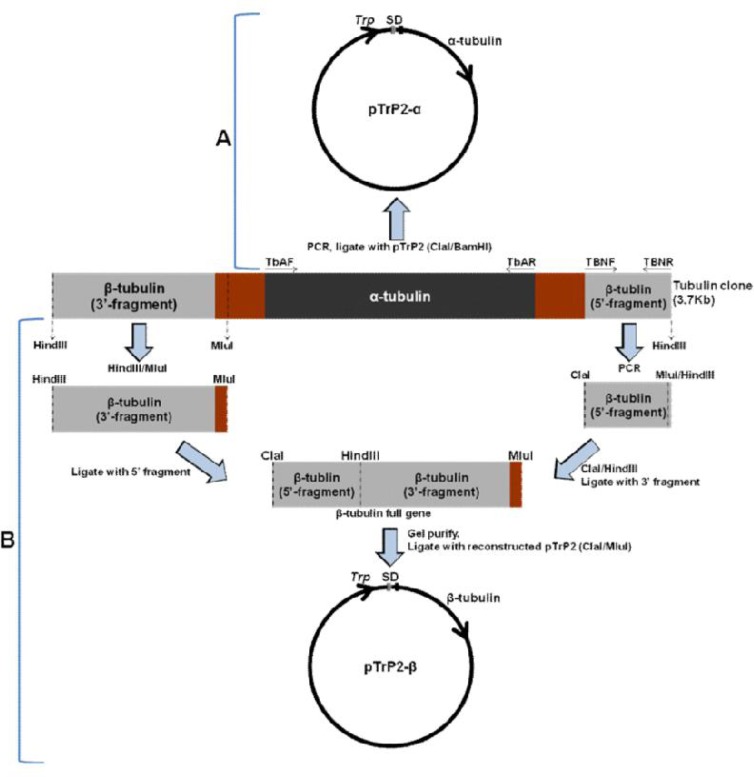
Schematic presentation of the salient features of *T. brucei* tubulin clone (3.7kb) in pBR322. Panel A shows how the α-tubulin (shaded black) was cloned; Panel B how the complete gene was generated from β-tubulin partial gene fragments (shaded grey). Depicted in brown are the inter-genic sequences between α- and β-tubulin genes (34). Dotted lines or arrows represent restriction enzyme sites while solid arrows represent primers used during PCR.

Two clones of *T. brucei brucei* α- and β-tubulin (19, 20) in pBR322 plasmid were provided by Prof. Thomas Seebeck, University of Basel. Each clone (∼3700bp) contained a full-length α-tubulin clone sandwiched by intergenic sequences, and two partial gene fragments of β-tubulin (Fig. 1).

The α-tubulin gene was PCR-amplified from pBR322 with specific forward and reverse primer:(TbAF,CCCAAGCTTATGCGTGAG GCTATCTGCATCCACATTG and TbAR, CGGGATCCCTAGTACTCCTCCACATC, containing restriction sites *Hind*III and *BamH*I respectively, underlined). The PCR products were digested with *Hind*III and *BamH*I and ligated with similarly digested pTrP2 (21, 22) (the expression vector used in this study) downstream the tryptophan (*Trp*) promoter and Shine-Dalgarno (SD).

### Construction of the full β-tubulin gene

The 3’ fragment (∼700bp) of the β-tubulin gene was released from pBR322 by digesting with *Hind*III and *Mlu*I and gel purified with Sephaglass gel purification kit (Amersham-Pharmacia Biotech). *Mlu*I cuts at position 227bp downstream of the transcription termination site for the β-tubulin gene (19, 20). The 5’ fragment (∼600bp) of the same gene was obtained by PCR with primers TbBNF: CCATCGATATGCGCGAAATCGTCTGCGTTCAGG, forward, and TbBNR: TCGCGAAGCTTCGAGATGAGC, reverse (Fig. 1). To facilitate cloning, the forward primer contained an artificial *Cla*I site (underlined) next to the start codon (ATG). On the other hand, the reverse primer contained the 11bp nucleotides (at the end of the beta tubulin gene) joined with the first 10bp nucleotides at the 3’ fragment (Fig. 1), as well as the *Hind*III cleavage site which naturally occurs at position 460bp of the full β-tubulin gene. The PCR product (5’ fragment, 600bp) was digested with *Cla*I and *Hind*III and ligated with the 3’ gene fragment (700bp). The ligation reaction was electrophoresed on a 1% agarose gel and the full β-tubulin gene purified with Sephaglass gel purification kit. The purified fragment (∼1300bp) was cloned into a re-constructed pTrP2 plasmid (pTrP2-β, see below and Fig. 1) via digestion with *Cla*I and *Mlu*I.

### Plasmid construction and cloning of the full length β-tubulin gene

With the exception of *Cla*I, the *T. brucei brucei* β-tubulin gene contains the restriction sites found in the pTrp2 cloning site (i.e. *Kpn*I, *Hind*III, *BamH*I and *Sal*I). Thus, the plasmid was re-constructed to introduce a unique cleavage site *Mlu*I by PCR-amplifying 600bp DNA from pBR322 with primers pBF: CCATCGATAGACGCAGACTCAGTACCTGAC, forward (with *Cla*I site) and pBR: CGGGATCCCGACGCGTTACTGCCTGACTC CCGTTGTTC, reverse (with *BamH*I and *Mlu*I sites). The PCR product was digested with *Cla*I and *Bam*HI and ligated with similarly digested pTrP2. The recombinant plasmid was isolated according to standard procedures and digested with *Cla*I and *Mlu*I to release the 600bp fragment. Then, following agarose gel electrophoresis, the vector fragment was gelpurified and ligated with the full-length β-tubulin gene (generated as outlined above, see Fig. 1), and transformed into *E. coli* for gene induction (see below).

After sub-cloning full-length tubulin genes into pTrP2, recombinant plasmids were designated pTrP2-α (containing the full-length *T. brucei* α-tubulin) and pTrP2-β (containing the full-length *T. brucei* β-tubulin). The clones were sequence-confirmed (ACGT, Wheeling, IL, USA) and compared with sequences in the GenBank, in which they were identical to those in genome data bases (98.2 - 99% identity) but with some single nucleotide polymorphisms (SNP) (approx. 16 per gene).

### Expression of trypanosome tubulin genes in E. coli

The expression plasmid pTrP2 contains a *Trp* promoter inducible by tryptophan starvation (22) or with an inducer 3-β-Indoleacrylic acid (IAA) (21). *T. brucei* α- and β-tubulin genes were individually expressed in *E. coli* as described by Yansura and Bass (21) using IAA. *E. coli* was freshly transformed with pTrP2-α and pTrP2-β following standard procedures (23, 24). Transformants were selected on Luria Bertani (LB) with agar, ampicillin (200µg/ml) and 25µg/mL L-tryptophan (to repress the promoter). Because expression levels can vary between strains (21) and culture volumes (25), first, we determined the ability of *E. coli* strains (DH5a, JM105, Top10 and BL21) to optimally express trypanosome tubulin by analysing expression in small-scale inductions (5ml) before undertaking large-scale inductions (500ml). Parallel controls included *E. coli* transformed with the parent plasmid (pTrP2) and a similar expression plasmid encoding *Onchocerca volvulas* β-tubulin (26).

Pellets from 1ml of overnight-induced cultures were analysed by SDS-PAGE as described by Yansura and Bass (21). To confirm the identity of the expressed tubulins, the separated proteins were trans-blotted onto nitrocellulose using the BioRad's mini-transblot system and probed with TAT1 and KMX, the monoclonal antibodies to *T. brucei* α-tubulin and *Physarum polycephalum* β-tubulin, respectively.

### Solubilisation and purification of the expressed trypanosome tubulin

The recombinant tubulins sequestered in *E. coli* inclusion bodies were purified as described by Sambrook et al, 1989 (23). Following induction, the *E. coli* pellet was homogenized in lysis buffer I (50mM Tris-Cl, pH 8.0, 1mM EDTA and 100mM NaCL) at 3mL per gram of cell pellet. Then, 40µL of 100mM PMSF (Phenylmethylsulphonyl fluoride) and 40µL 100mg/mL of lysozyme were added to the lysate and incubated on ice for 30min with occasional stirring. To disrupt the resultant spheroblasts, deoxycholic acid was added at 4mg per gram of cell pellet while stirring with a plastic pipette and incubated at 37 °C till the lysate became viscous. Then, DNase1 was added (3µL of 1mg/mL) and the mixture incubated at room temperature for 20min, centrifuged at 12000g for 20min and the pellet homogenized in lysis buffer II (50mM Tris-Cl, pH 8.0, 10mM EDTA, 100mM NaCl and 0.5% Triton X-100) at 9 volumes per gram of cell pellet. After incubating at room temperature for 15min, the lysate was centrifuged at 12000g for 20min and the pellet washed again with lysis buffers (I and II), and centrifuged to obtain the final pellet which was suspended in 3mL of 8M urea with 100mM PMSF.

After incubating for an additional hour at room temperature, the urea-protein solution was diluted 20-fold with alkaline buffer (50mM KH2PO4, pH 10.7, 1mM EDTA and 50mM NaCl), adjusted to pH 10.7 and incubated on ice for 30min. Then, the pH was adjusted to 8.0 and the solution incubated on ice for an additional 30min followed by centrifugation at 12000g. The supernatant containing tubulin was concentrated to 1/3^rd^ of the original volume using amicon cones (45Da). The concentrate was diluted 3-fold with MES buffer (0.085M MES, pH 6.0, 1mM EGTA, 0.1mM EDTA and 0.5mM MgSO_4_) and concentrated again. Portions of α- and β-tubulins were also mixed and co-solubilized together in urea as α/β-tubulin mixture.

### Dialysis

The urea-dissolved tubulin was dialyzed against MES buffer containing 0.1M PMSF and 2M glycerol in 45Da dialysis tubing as described elsewhere (27). After dialysis, the protein concentrate was analyzed with SDS-PAGE and stored at –80°C. Protein concentration was determined by the Bradford assay using bovine serum albumin as a standard.

### Immunization of rabbits

Prior to immunization, the recombinant tubulins were extensively dialyzed against phosphate buffered saline (PBS) with 100µM PMSF to neutralize MES buffer, and diluted to final concentration of 450µg/mL, which was used as immunization dose. The immunogens were; α, β, and α/β mixture while the control was PBS. These (i.e., α, β, α/β and PBS) were emulsified with 1ml of Freund's complete adjuvant using multiple passages through a 20 gauge needle till the solution became thick and creamy. Four groups of rabbits (each with four animals) per immunogen were employed. Pre-immune sera (PIS) were collected from the animals before immunizations. Then, 500µl of the emulsified immunogens or PBS were injected subcutaneously into the rabbits as described elsewhere (28). Three booster doses (in Freund's incomplete adjuvant) were administered. Test bleeds for sera preparation occurred after two, eight and eleven weeks.

### Growth Inhibition Assays

The *T. brucei* bloodstream form (strain UTRO 010291B) was grown as described previously (29) in supplemented MEM (Minimum Essential Medium Gibco) at 37°C, 5% CO_2_ in a humidified CO_2_ incubator. Growth inhibition assays were performed as described previously (16, 30) in 100µl of medium with serial dilutions of up to 25% antisera in 96 well-tissue culture plates (Corning, New York, USA). Parasites were seeded at 2 x 10^5^ cells mL^-1^ per well in triplicate wells for every antisera batch. For comparison, growth inhibition assays involving monoclonal antibodies to *T. brucei* α- and β-tubulin were also performed. Control wells contained culture medium alone (no anti-serum), culture medium with similar dilutions of pre-immune serum (PIS) and sera from control rabbits (immunized with PBS and adjuvant). Cells were incubated at 37 °C, 5% CO_2_, for 24h and counted using a Neuber improved hemocytometer.

### Ethical considerations

This study was approved by the Research and Higher degrees committee of the Faculty of Veterinary Medicine, Makerere University. Rabbits were kept in the animal house facility of the School of Veterinary Medicine under hygienic conditions and were provided veterinary care. Rabbits were handled humanely according to standard procedures (28). After the study, all rabbits were clinically examined to ensure absence of disease symptoms and none was euthanized.

## Results

### T. brucei brucei α and β tubulin genes were successfully expressed in E. coli

**Fig. 2:**
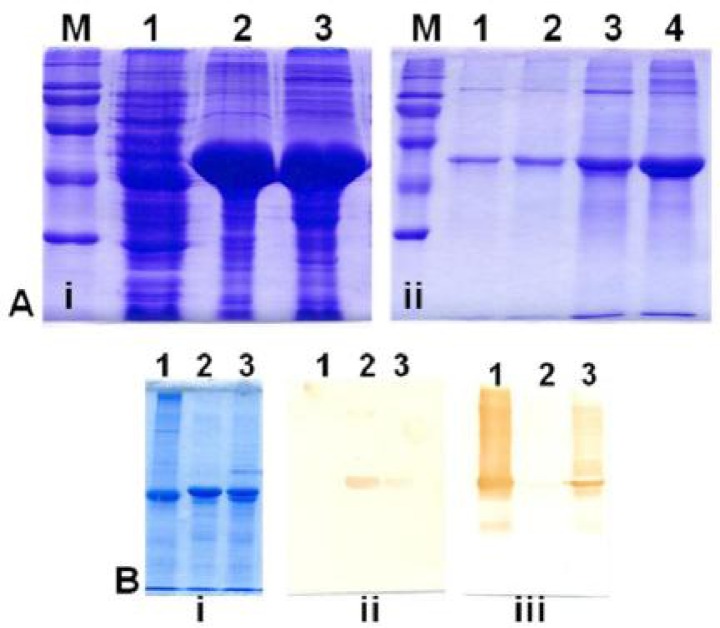
Expression of *T. brucei* α- and β-tubulin genes in *E. coli*. **Panel A(i):** M, MW marker; 1, bacterial lysates of induced cultures transformed with only pTrp2; 2 and 3 proteins in bacterial lysates induced for expression of α- and β-tubulin, respectively. **Panel A(ii):** enriched isolation of *T. brucei* α- and β-tubulin with amicon cones; M, MW marker; 1, *Onchocerca* β-tubulin; 2, α-tubulin; 3, β-tubulin; 4, α/β-tubulin mixture. **Panel B:** B(i), Coomassie blue stained gel; B(ii) and B(iii), similar gel as in B(i) probed with KMX and TAT1 monoclonal antibodies, respectively. Lanes 1, α-tubulin; 2, β-tubulin; 3, α/β-tubulin mixture.

*T. brucei* α- and β-tubulin genes were successfully expressed in *E. coli* (Fig. 2) with the four strains (BL21, DH5a, JM105 and Top10) expressing genes to varying detectable levels.

The β-tubulin gene was optimally expressed in all the four strains but best in DH5a while the α-tubulin was optimally expressed in only JM105.

Similar to the native *T. brucei* tubulin isoforms (31), the molecular weights (MW) for the bacterially expressed tubulins were identical (∼50kDa) and indistinguishable by gel migration. Furthermore, from sequence data of the cloned genes, the predicted MW for these proteins were also nearly identical (∼49673.0195Da vs. 49448.6367Da, for α- and β-tubulin respectively) and corresponded to that predicted for *Onchocerca* β-tubulin (i.e. 50119.296 Da (26)). The concentrated tubulins yielded ∼0.7g protein per litre of culture at approx. 70% purity. The identity of the bacterially expressed tubulin was confirmed by strong reaction with specific monoclonal antibodies TAT1 and KMX which did not react with the opposite tubulin (Fig. 2, panel B).

### T. brucei brucei recombinant tubulin stimulated high levels of polyclonal antibodies in rabbits

**Fig. 3:**
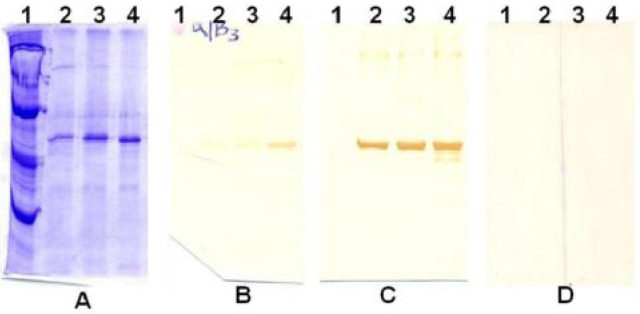
Production of antibodies in rabbits immunized with bacterially expressed tubulin. **Panel A:** Coomassie Blue stained gel. **Panels B & C:** similar gels as in A blotted and probed with 2^nd^- and 3^rd^-bleed antisera. **Panel D:** similar gels as in A probed with pre-immune sera or third bleed adjuvant antisera (controls). Lanes: 1, MW marker; 2, α-tubulin; 3, β-tubulin; 4, α/β-tubulin mixture. Antisera batches from rabbits individually immunized with α- or β-tubulin gave similar results depending on the tubulin isoform used. Signal intensity was stronger with each successive antigen boost (Panel C)

Antibodies were successfully produced in all the rabbits immunized with the recombinant tubulins and high titres were achieved upon screening with ELISA (not shown) and western blotting (Fig. 3, panels B & C. Rabbit anti-sera to recombinant tubulins also reacted with native tubulin in native *T. brucei* homogenates that were electrophoresed and transblotted alongside the bacterially expressed tubulins (not shown).

### The effect of polyclonal antibodies to recombinant tubulin on T. brucei brucei growth

The lowest antisera dilutions (25%) from rabbits immunized with recombinant tubulins inhibited trypanosome growth (i.e. in comparison with controls –adjuvant, and pre-immune sera). The most cytotoxic antisera were from a rabbit immunized with a mixture of both alpha and beta tubulins (rα/β mixture). Table 1 shows antisera batches from this rabbit (α/β3).

**Table 1:** Inhibition of *T. brucei* bloodstream form (x10^4^/mL) in culture medium with antisera from rabbits immunized with bacterially expressed trypanosome tubulin

Antisera	No. antisera (controls)	Antisera Dilution (v/v) and cell counts				
		1:10	1:20	1:40	1:80	1:160
Rα1	270	187	207	236	189	201
Rα2	308	120	267	258	202	243
Rα3	301	147	277	301	211	287
Rα4	303	158	287	287	234	245
Rβ1	299	155	256	278	245	298
Rβ2	298	161	276	319	214	289
Rβ3	308	157	298	298	250	320
Rβ4	304	158	265	317	267	302
Rα/β1	297	179	287	319	243	287
Rα/β2	311	158	256	300	253	290
**Rα/β3**	**307**	**000**	**007**	**107**	**233**	**235**
Rα/β4	301	178	298	328	287	345
**Rα/β3PIS**	**321**	**325**	**385**	**355**	**233**	**287**
RAdj1	315	157	276	298	219	276
RAdj2	299	144	298	321	236	276
RAdj3	368	181	286	301	190	297

*T. b. brucei* bloodstream form was inhibited by the least diluted (25%) 3^rd^ bleed antisera, but higher dilutions were inefficient. The exception was the antiserum from a rabbit immunized with Rα/β3 mixture (in bold face). Rα/β3, antiserum to a mixture of *T. b. brucei* recombinant α- and β- tubulins; Rα3, antiserum to *T. b. brucei* α-tubulin; Rβ3, antiserum to *T. b. brucei* β-tubulin; Rα/β3PIS, Pre-Immune Serum from the rabbit producing cytotoxic antiserum; RAdj, RAdj2 and RAdj3, sera from rabbits immunized with Freund's adjuvant alone. Each data point represents mean results from three experiments

Interestingly, the pre-immune serum from the same rabbit did not inhibit trypanosome growth, implying the effect was due to immunization with recombinant tubulin. However, attempts to precipitate the killing antibody through immunopreciptation experiments were futile. Furthermore, it was disappointing that similar results were not reproduced in other rabbits. Indeed for most rabbits, there was no apparent effect on trypanosome growth at higher antisera dilutions. This may imply that bacterially expressed trypanosome tubulin is not as effective as the native one at producing trypano-destructive antibodies in rabbits.

## Discussion

In this study, the bacterially expressed trypanosome tubulin was not as effective at producing cyto-toxic antibodies in rabbits as the native tubulin (16). While the most obvious reason for lack of cytotoxicity is failure of bacterially expressed tubulin to achieve proper folding, it was surprising that the *T. brucei* tubulin-monoclonal antibodies raised in other laboratories were also ineffective at inhibiting trypanosome growth. This discouraged further pursuits for properly folded tubulin such as expression in eukaryotic systems. Moreover, recent studies with recombinant tubulins also reported weak protection of mice immunized with recombinant tubulin isoforms (8, 9, 18). As such, the subtle immunoprotective effects with most anti-trypanosome antigens targeting the humoral defence mechanism have been attributed to initial nonspecific mechanisms in the host but not memory (8). Trypanocidal components in mammalian serum have been well documented in killing trypanosmes; IgM and other antibody independent mechanisms can mediate trypanolysis in infected mammals (11, 32).

The fact that African trypanosomes induce B-cell apoptosis makes it difficult for the infected host to mount an antibody mediated immune response (8, 33). In the study of Radwanska et al (33), it's reiterated that in experimental immunization models, any subtle protection following infection is attributed to IgM or the initial host response but not immunological memory (8, 33). Explicitly, a positive outcome may be a result of the immediate effect of immune modulation by the vaccine boost (8). As such, current and future anti-trypanosome vaccine designs should put more emphasis on targets other than the humoral defence mechanisms, such as blocking early initial stages in the host as well as vector stages (8).

On the other hand, presence of unknown factors unrelated with tubulin in the immunogen preps may also cause immunoprotection or cytotoxic effects particularly in previous studies (9, 15, 16, 18). Since it is notoriously difficult to make a biochemical preparation of a protein that is absolutely pure, it is possible that the interesting and encouraging results in previous work were related to unidentified molecule in the trypanosome tubulin preparations.

In conclusion, bacterially expressed trypanosome tubulin is not as effective as native trypanosome tubulin at producing cytotoxic antibodies to kill bloodstream trypanosomes in culture.
